# Supplementary material for: Rapid quantification assay of hepatitis B virus DNA in human serum and plasma by Fully Automated Genetic Analyzer μTASWako g1
Source: PLoS One. 2023 Feb 9;18(2):e0278143. doi: 10.1371/journal.pone.0278143 (PMC9910706; doi:10.1371/journal.pone.0278143)
Supplement: S1 Table — Values shown are median (interquartile range). (DOCX) [file pone.0278143.s001.docx]

**S1 Table. Background information of clinical samples from patients with HBV infection for correlation study (n = 207)**. Values shown are median (interquartile range).
