# Supplementary material for: Rapid quantification assay of hepatitis B virus DNA in human serum and plasma by Fully Automated Genetic Analyzer μTASWako g1
Source: PLoS One. 2023 Feb 9;18(2):e0278143. doi: 10.1371/journal.pone.0278143 (PMC9910706; doi:10.1371/journal.pone.0278143)
Supplement: S2 Table — (DOCX) [file pone.0278143.s002.docx]

**S2 Table. The comparison of discrepancy between CAP/CTM v2 and μTASWako g1 assay in individual samples with lower HBV titer.**
