# Supplementary material for: Rapid quantification assay of hepatitis B virus DNA in human serum and plasma by Fully Automated Genetic Analyzer μTASWako g1
Source: PLoS One. 2023 Feb 9;18(2):e0278143. doi: 10.1371/journal.pone.0278143 (PMC9910706; doi:10.1371/journal.pone.0278143)
Supplement: S3 Table — (DOCX) [file pone.0278143.s003.docx]

**S3 Table.　Comparison of assay specification of CAP/CTM v2 assay, Abbott RealTime HBV assay and Aptima HBV Quant assay.**

* Limit of Detection

# Lower Limit of Quantification
