# Supplementary material for: Rapid quantification assay of hepatitis B virus DNA in human serum and plasma by Fully Automated Genetic Analyzer μTASWako g1
Source: PLoS One. 2023 Feb 9;18(2):e0278143. doi: 10.1371/journal.pone.0278143 (PMC9910706; doi:10.1371/journal.pone.0278143)
Supplement: Attachment — Submitted filename: Response to Reviewers.docx [file pone.0278143.s004.docx]

**Dear Academic editor and reviewer,**

I would like to provide following answer/comment to your question/comment on the manuscript.

**To Academic Editor**

**1. Please ensure that your manuscript meets PLOS ONE's style requirements, including those for file naming. The PLOS ONE style templates can be found at**

[**https://journals.plos.org/plosone/s/file?id=wjVg/PLOSOne_formatting_sample_main_body.pdf**](https://journals.plos.org/plosone/s/file?id=wjVg/PLOSOne_formatting_sample_main_body.pdf)

**and**

[**https://journals.plos.org/plosone/s/file?id=ba62/PLOSOne_formatting_sample_title_authors_affiliations.pdf**](https://journals.plos.org/plosone/s/file?id=ba62/PLOSOne_formatting_sample_title_authors_affiliations.pdf)

**Answer**

We have confirmed that our manuscript meets PLOS ONE’s Style requirement.

**2. We note that the grant information you provided in the ‘Funding Information’ and ‘Financial Disclosure’ sections do not match.**

**When you resubmit, please ensure that you provide the correct grant numbers for the awards you received for your study in the ‘Funding Information’ section.**

**Answer**

Some of authors are belong to company (Fujifilm Wako Pure Chemical), so I thought that I should put the information that the authors have salary from the company. Actually, the company did not fund grant for the study. So, there is not any grant numbers for this study.

**3. Thank you for stating the following in the Financial Disclosure section:**

**"Specific grant numbers; None**

**Initials of authors who received each award; M.W**

**Full names of commercial companies that funded the study or authors; Fujifilm Wako Pure Chemical Corporation**

**Initials of authors who received salary or other funding from commercial companies; M.W and T.K**

**URLs to sponsors’ websites; https://www.fujifilm.com/ffwk/en**

**The funders had role in decision to publish of the manuscript."**

**We note that one or more of the authors have an affiliation to the commercial funders of this research study: Fujifilm Wako Pure Chemical Corporation**

**(1) Please provide an amended Funding Statement declaring this commercial affiliation, as well as a statement regarding the Role of Funders in your study. If the funding organization did not play a role in the study design, data collection and analysis, decision to publish, or preparation of the manuscript and only provided financial support in the form of authors' salaries and/or research materials, please review your statements relating to the author contributions, and ensure you have specifically and accurately indicated the role(s) that these authors had in your study. You can update author roles in the Author Contributions section of the online submission form.**

**Please also include the following statement within your amended Funding Statement.**

**“The funder provided support in the form of salaries for authors [insert relevant initials], but did not have any additional role in the study design, data collection and analysis, decision to publish, or preparation of the manuscript. The specific roles of these authors are articulated in the ‘author contributions’ section.”**

**If your commercial affiliation did play a role in your study, please state and explain this role within your updated Funding Statement.**

**(2) Please also provide an updated Competing Interests Statement declaring this commercial affiliation along with any other relevant declarations relating to employment, consultancy, patents, products in development, or marketed products, etc.**

**Within your Competing Interests Statement, please confirm that this commercial affiliation does not alter your adherence to all PLOS ONE policies on sharing data and materials by including the following statement: ""This does not alter our adherence to PLOS ONE policies on sharing data and materials.” (as detailed online in our guide for authors http://journals.plos.org/plosone/s/competing-interests).**

**If this adherence statement is not accurate and there are restrictions on sharing of data and/or materials, please state these. Please note that we cannot proceed with consideration of your article until this information has been declared.**

**Please include both an updated Funding Statement and Competing Interests Statement in your cover letter. We will change the online submission form on your behalf.**

**Answer**

Moto Watanabe and Tomohisa Kawabata are belong to Fujifilm Wako Pure Chemical Corporation. However, Fujifilm Wako Pure Chemical did not play a role in the study design, data collection and analysis, decision to publish, or preparation of the manuscript and only provided MW and TK’s salaries and research materials.

So, I will revise the funding statement as following in the

“The funder provided support in the form of salaries for authors M.W and T.K, but did not have any additional role in the study design, data collection and analysis, decision to publish, or preparation of the manuscript. The specific roles of these authors are articulated in the ‘author contributions’ section.”

I will also declare following sentence,

This does not alter our adherence to PLOS ONE policies on sharing data and materials.

**4. PLOS requires an ORCID iD for the corresponding author in Editorial Manager on papers submitted after December 6th, 2016. Please ensure that you have an** **ORCID iD and that it is validated in Editorial Manager. To do this, go to ‘Update my Information’ (in the upper left-hand corner of the main menu), and click on the Fetch/Validate link next to the ORCID field. This will take you to the ORCID site and allow you to create a new iD or authenticate a pre-existing iD in Editorial Manager. Please see the following video for instructions on linking an ORCID iD to your Editorial Manager account:**

**https://www.youtube.com/watch?v=_xcclfuvtxQ**

**Answer**

I will submit my ORCID iD.　 **0000-0002-5305-1392**

**5. Please include your full ethics statement in the ‘Methods’ section of your manuscript file. In your statement, please include the full name of the IRB or ethics committee who approved or waived your study, as well as whether or not you obtained informed written or verbal consent. If consent was waived for your study, please include this information in your statement as well.**

**Answer**

We have revised manuscript to include the name of IRB. Written informed consent was waived due to the retrospective use of the stored serum samples.

**6. Please review your reference list to ensure that it is complete and correct. If you have cited papers that have been retracted, please include the rationale for doing so in the manuscript text, or remove these references and replace them with relevant current references. Any changes to the reference list should be mentioned in the rebuttal letter that accompanies your revised manuscript. If you need to cite a retracted article, indicate the article’s retracted status in the References list and also include a citation and full reference for the retraction notice.**

**Answer**

We have confirmed the reference list. No retracted article was listed. We have made minor change to omit issue number and corrected typo. The changes are marked-up on the manuscript.

**To Reviewer**

**Question 1:**

**In the methods section, authors described as follows: “Afterward, 33 PCR cycles with a longer extension (70 s at 73.5°C) was performed with one CE injection from the PCR chamber for each subsequent cycle to analyze the accumulated target amplicons and took 56 min to complete the entire qPCR-CE step.” How did you sample from the PCR chamber into the electrophoresis channel?**

**Answer:**

PCR product (DNA) is moved from PCR chamber toward electrophoresis channel by applying electrical current (0.2 µA) to PCR chamber via two microchannels which filled with a reagent for electrophoresis. The reagent wells were physically separated from electrode contact wells to prevent instrument contamination from reagent and amplicons. Electrical connection between the two wells was made through printed carbon electrodes on the chip. The detail of microfluidic chip design and the movement of DNA during the CE were well illustrated in previous research article (reference#18). The article is also helpful to understand how it works.

**Question 2:**

**Normally in PCR reactions, it is thought that the amplification reaction efficiency changes with changes in the concentration of the multiplex partner when the reaction is multiplexed, but what is the reason why the IC reactivity did not change when the HBV virus concentration was changed?**

**Answer:**

There are three reasons why the IC reactivity did not change when the HBV virus concentration was changed.

1. In the µTASWAKO g1 HBV assay, IC DNA (approximately 1,200 copies/assay) was added to the sample at the beginning of the DNA purification step, therefore, the IC DNA concentration in PCR mixture is almost consistent every times.

2. IC DNA was designed so that IC target sequence can be amplified by HBV primers. Basically, the PCR amplification efficiency of IC were equivalent to that of HBV target due to the same primer performance.

3. The IC DNA concentration, the primer concentration, and IC detection threshold for qPCR-CE have been optimized to reproducibly detect IC Cq by testing the samples containing high concentration of HBV (~3.5×10^8 IU/mL). The balance of those three factors is most important to enable IC detection without interference by HBV-amplification even when 3.5×10^8 IU/mL of HBV sample tested. Primer consumption in a reaction-mixture results in reduction of amplification efficiency as a progressing of PCR cycles. In the case of co-amplification of HBV (3.5×10^8 IU/mL) and IC, the HBV primers were consumed, however, the remaining primers maintains IC-amplification efficiency until PCR cycle (at 26-27) when IC-amplicons exceed the detection threshold for qPCR-CE. We have confirmed that IC Cq does not change across dynamic range of HBV quantification (~3.5×10^8 IU/mL), while IC Cq may change when testing of the sample containing HBV more than 10^9 IU/mL. If the assay detected HBV DNA at over dynamic range, the sample will need to be diluted with HBV-negative sample for re-test.

**Question 3:**

**How can this "quick results" measurement method be useful in real-world clinical practice? Please discuss this point.**

**Answer:**

HBV is potentially reactivated in some patients who undergo chemotherapy or immunosuppressive therapies and HBV reactivation often causes life-threatening liver failure. Reactivation occurs not only in HBsAg-seropositive patients but also in those with resolved HBV infection who are seronegative for HBsAg but seropositive for antibody against hepatitis B core antigen (anti-HBc) and/or antibody against HBsAg (anti-HBs). To prevent HBV reactivation-related hepatitis, HBV DNA monitoring-guided preemptive antiviral therapy using anti-HBV nucleos(t)ide analogs (NAs) is recommended by several guidelines for patients with resolved HBV infection, and it is mandatory to detect the emergence of serum HBV DNA as early as possible to start anti-HBV therapy early and effectively. However, the duration required for HBV DNA measurements, including both qualitative and quantitative, hinders agile and effective preventive strategies in real clinical practice. HBV DNA measurement technology with rapidly available results will be a strong advantage for monitoring patients at risk of HBV reactivation since the “quick results” possibly contribute to minimal delay in clinical decision-making after blood collection from the patients.

Best regards,

Tomohisa Kawabata
