# Supplementary material for: Rapid quantification assay of hepatitis B virus DNA in human serum and plasma by Fully Automated Genetic Analyzer μTASWako g1
Source: PLoS One. 2023 Feb 9;18(2):e0278143. doi: 10.1371/journal.pone.0278143 (PMC9910706; doi:10.1371/journal.pone.0278143)
Supplement: Attachment — Submitted filename: PONE_D_22_30604R1_Comments.docx [file pone.0278143.s005.docx]

Comments to the Authors

I have carefully reviewed your replies. You have responded to all questions appropriately. I did not think any further revision was necessary.
